# Mutations in *CCNO* and *MCIDAS* lead to a mucociliary clearance disorder due to reduced generation of multiple motile cilia

**DOI:** 10.1186/2194-7791-2-S1-A15

**Published:** 2015-07-01

**Authors:** J Wallmeier, M Boon, D Al Mutairi, NT Loges, L Ma, C-T Chen, H Olbrich, P Pennekamp, T Menchen, G Dougherty, C Werner, M Jaspers, M Griese, E Horak, C Körner-Rettberg, S Schmitt-Grohé, T Zimmermann, A Hevroni, R Abitbul, A Avital, R Soferman, I Amirav, H Mitchison, M Jorissen, F Alkuraya, C Kintner, H Omran

**Affiliations:** General Pediatrics, University Hospital Münster, Münster, Germany; Department of Pediatrics, Pediatric Pulmonology, University Hospital of Leuven, Leuven, Belgium; Department of Pathology, Faculty of Medicine, Health Sciences Center, Kuwait University, Safat, Kuwait; Molecular Neurobiology Laboratory, The Salk Institute for Biological Studies, San Diego, CA USA; Department of Otorhinolaryngology, Head & Neck Surgery, University Hospital Leuven, Leuven, Belgium; Department of Pediatric Pulmonology, Hauner Children's Hospital, Member of the German Center for Lung Research (DZL), Ludwig Maximilians University, Munich, Germany; Department of Pediatrics and Adolescents, Division of Cardiology and Pulmonology, Innsbruck Medical University, Innsbruck, Austria; Department of Pediatrics and Adolescent Medicine, St. Josef Hospital, Ruhr-Universität Bochum, Bochum, Germany; Department of Pediatrics, Pediatric Pulmonology, University Hospital Bonn, Bonn, Germany; Department of Pediatrics, Pediatric Pulmonology, University Hospital, Erlangen, Germany; Institute of Pulmonology, Hadassah-Hebrew University Medical Centers, Jerusalem, Israel; Department of Pediatric, Ziv Medical Center, Faculty of Medicine, Bar IIan University, Safed, 13100 Israel; Department of Pediatric Pulmonology, Critical Care and Sleep Medicine, Dana Children's Hospital, Tel Aviv Sourasky Medical Center, 6 Weizman Street, Tel Aviv, 64239 Israel; Molecular Medicine Unit, Birth Defects Research Centre, Institute of Child Health, University College London, London, WC1N 1EH UK; Department of Genetics, King Faisal Specialist Hospital and Research Center, Riyadh, Saudi Arabia; Department of Anatomy and Cell Biology, College of Medicine, Alfaisal University, Riyadh, Saudi Arabia

## Meeting abstract

Since the 1980s, a few case reports described patients with oto-rhino-pulmonary symptoms and respiratory epithelia lacking cilia who were subsequently diagnosed to suffer from "ciliary aplasia" or "acilia syndrome". Via a whole exome sequencing approach, we analyzed "ciliary aplasia" candidates (including patients from previous reports) and identified recessive mutations in *CCNO* (encoding Cyclin O) and *MCIDAS* (encoding Multicilin) in 9 and 16 individuals, respectively [1, 2]. All individuals suffered from severe respiratory symptoms of the upper and lower airways and development of bronchiectasis at an early age. Thorough analysis of respiratory epithelial cells obtained by nasal brush biopsy by both transmission electron microscopy (TEM) and immunofluorescence analysis (IF) revealed that respiratory cilia were not completely absent; some cells still retained one or two cilia. These cells not only showed a reduction of cilia by TEM and IF, but also a reduction and mislocalization of basal bodies and rootlets throughout the cytoplasm. Detailed analyses by IF in both man and *Xenopus* revealed that this reduction of cilia number was due to a centriole amplification defect in the acentriolar pathway, which is specific for multiciliated cells [1, 2].

IF showed that MCIDAS functions upstream of CCNO and FOXJ1, which is important for transcriptional control of axonemal motor proteins such as DNAH5 and CCDC39. Whereas cilia in *CCNO*-mutant cells still contain motility-related proteins such as DNAH5 and CCDC39 and can display normal beating patterns, *MCIDAS*-mutant cells are immotile and lack those axonemal motor proteins [1, 2] .

*MCIDAS* and *CCNO* lie adjacently on chromosome 5q11 in a region related to multiciliogenesis, and act in the same pathway underlying multiciliogenesis. Based on these findings, we propose that this disease now should be referred to as "mucociliary clearance disorder with reduced generation of multiple motile cilia" (RGMC).
